# Robotic assisted hysterectomy and colpectomy as gender affirming surgery in trans men: an instructional video and perioperative considerations

**DOI:** 10.3389/fsurg.2025.1608666

**Published:** 2025-07-31

**Authors:** Tilemachos Kavvadias, Emma Garcia Gosch, Viola Heinzelmann-Schwarz

**Affiliations:** ^1^ Innovation Focus on Gender Variance, University Hospital of Basel, Basel, Switzerland; ^2^ Clinic for Gynecology and Gynecologic Oncology, University Hospital of Basel, Basel, Switzerland

**Keywords:** transgender, robotic assisted colpectomy, instructional video, gender affirming surgery, perioperative complications

## Abstract

**Background/objectives:**

The objective of this paper is to present a method of hysterectomy and colpectomy in trans men seeking gender affirming surgery using a uterovaginal manipulator device.

**Methods:**

Eighteen consecutive patients underwent robotic assisted laparoscopic hysterectomy and colpectomy as gender affirming surgery. The perioperative descriptive statistical data as well as complications were documented. An instructional video has been prepared and is provided as Supplementary Material.

**Results:**

Patients had a median age of 28 years and a mean BMI of 23.5 ± 3.8 Kg/m^2^. Mean operating time was 175 ± 25 min (median 180), mean blood loss was 219 ± 142 ml (median 200) and mean hospital stay was 4.6 ± 5.9 days (median 3). Two major (one compartment syndrome which required re-surgery and one bladder injury which resolved intraoperatively) and seven minor complications occurred (5 patients with urinary retention and 2 urinary tract tract infections) occurred.

**Conclusions:**

Robotic assisted laparoscopic hysterectomy and colpectomy in trans men seeking gender affirming surgery is a feasible option, facilitated by the use of a uterovaginal manipulator device. Candidates for the procedure should be properly counseled in anticipation of possible complications.

## Introduction

The number of people seeking Gender Affirming Surgery (GAS) is rising worldwide, following the rise in the number of people who identify as transgender (1). The removal of the female genitalia can include hysterectomy, salpingo-oophorectomy and colpectomy, alone or a combination thereof. While hysterectomy and salpingo-oophorectomy are quite standardized procedures, colpectomy is regarded as a complex surgical intervention, as it requires sufficient knowledge of the pelvic anatomy and meticulous dissection of delicate structures and organs that surround the vagina, such as the bladder, ureter and rectum. Moreover, colpectomy is associated with serious perioperative and postoperative complications, such as organ or vascular injury, bleeding and hematoma as well as fistula formation (2).

Unfortunately, international literature is lacking studies that assess the feasibility and improvement of colpectomy methods and instructional articles and videos are scarce. This paper is an attempt to describe the method of performing colpectomy as part of a gender affirming surgical procedure, using a uterovaginal manipulator device, for educational purposes.

## Materials and methods

Data from trans men who underwent robotic assisted laparoscopic hysterectomy and colpectomy in our institution between November 2022 and February 2025 were prospectively collected. All transgender people who seek surgery in our institution are being evaluated by an interdisciplinary team of gynecological, urological and plastic surgeons as well psychiatrists and psychologists. Written informed consent to participate in this study was obtained from all participants.

### Surgical procedure

#### Preoperative considerations

In our institution the procedure is approached laparoscopically with the use of the DaVinci Xi Ⓡ robotic system (Intuitive Surgical), but the method can be applied on conventional laparoscopy as well. We use the Schaer uterovaginal manipulator, which can be used in various applications besides the colpectomy, such as supracervical hysterectomy and sacrocolpopexy (3). No bowel preparation is needed, although this can vary according to different protocols in various institutions. All counseling and work up is being performed in the outpatient clinic and the patient is administered on the day of surgery.

#### Method of surgery

**Figure 1:**
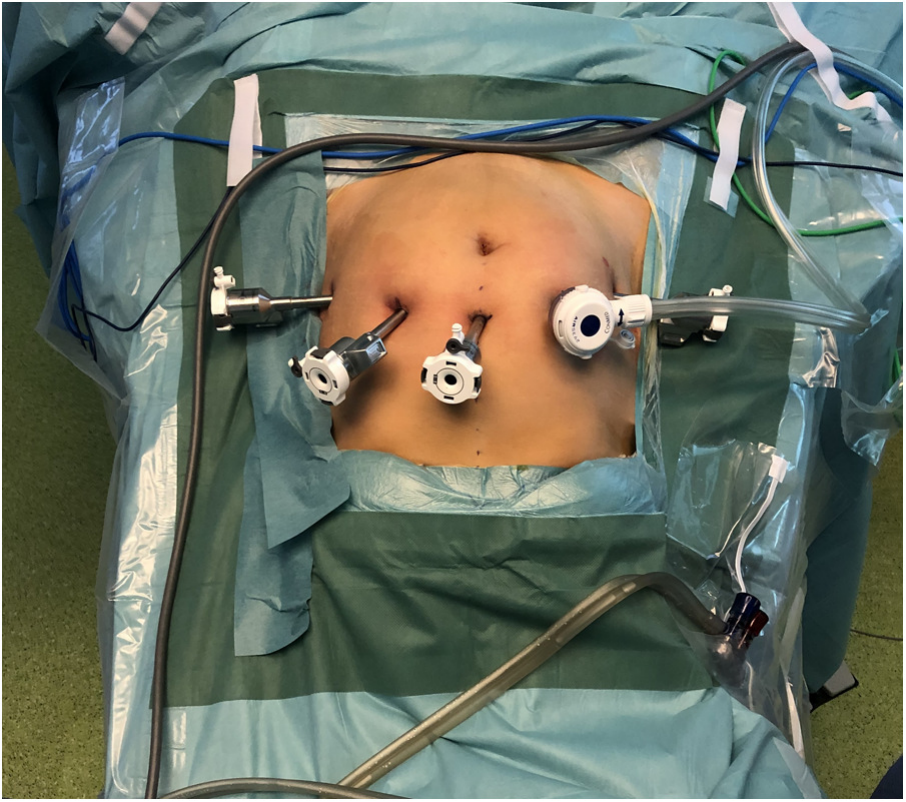
Trocar placement of the daVinci Xi robotic system.

We begin the laparoscopy entering the abdomen with a needle through an infraumbilical incision and introducing the pneumoperitoneum. The camera trocar is placed 5 cm above the umbilicus. Three additional robotic trocars and one conventional trocar are placed along a semicircular line around the umbilicus (Figure 1). The intra abdominal pressure is set between ten and fourteen mmHg according to the patient's stature and after agreement with the anesthesiologist.

**Figure 2:**
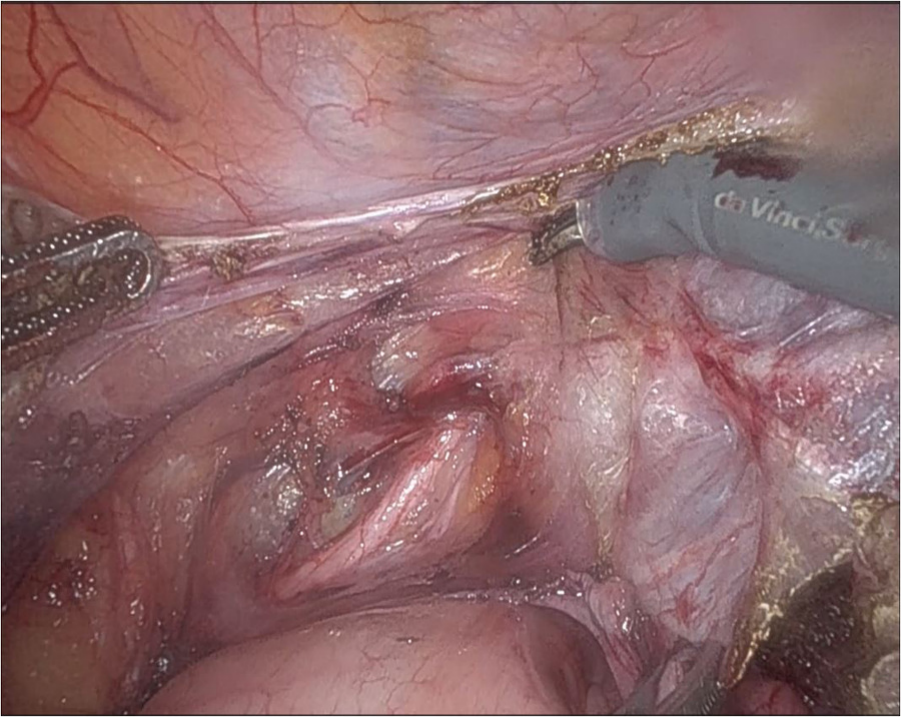
The left ureterovaginal junction. The ureteric path is clearly dissected from the uterine artery.

Hysterectomy is performed in a usual manner, while additional caution is being paid on the opening of the retroperineal space after cutting of the round ligament. At this point we dissect the paravesical and pararectal space as thoroughly as possible, so that the ureter is free from the periureteral connective tissue and visible, at least until crossing the uterine artery, better—if possible—until the ureterovesical junction (Figure 2).

The next step involves the dissection of the vesicovaginal space. After the opening of the supravaginal septum (vesicovaginal ligament) the space between bladder and anterior vaginal wall is entered and dissected using mostly blunt dissection or monopolar scissors where needed. Note how the Schaer manipulator helps in moving the cervix dorsally, but also—because of its flat anterior surface—in providing an undelay to dissect the vesicocaginal space. We continue until the lower vaginal part is reached, directly under the folley catheter balloon which can be located easily. Sometimes, mostly in patients with previous surgery, filling the bladder with 100–150 ml saline solution can be helpful in identifying the bladder. In this step it is important that the surgeon stays strictly on the anterior part of the vagina and leaves the lateral attachments (paracolpium) uncompromised to avoid unnecessary bleeding. At this point, with the ureter being lateralized through dissection and also through the ‘pushing forward’ on the Schaer manipulator as well as with the bladder completely dissected from the vagina, the dissection of the broad ligament is completed and the uterine artery can be ligated using a clip or coagulation.

The next step involves the dissection of the uterosacral ligaments, which can be easily identified by moving the Schaer manipulator anteriorly. Then, the rectovaginal space is dissected, after creating tension on the parietal peritoneum cranially of the rectum. The posterior vaginal wall is being separated from the rectum. Again, as in the anterior part of the vagina, the flat posterior surface of the manipulator device offers an excellent view of the correct plane. When the posterior part of the vagina is completely dissected, we move cranially again and complete the dissection of the cardinal ligament. Sufficient cranial pressure on the manipulator device and having previously dissected the uterine artery and the ureter we can move closely to the vaginal surface in order to always keep a safe distance from ureter, which passes anteriolaterally to the cardinal ligament on each side.

Finally, the lateral attachments of the vagina are being dissected. This can be performed either using clips or the bipolar forceps. The form of the Schaer manipulator facilitates the sliding of the forceps on the connective tissue between the anterior and the posterior vaginal wall, making sure that only the vaginal epithelium is being dissected, whilst the Halban fascia remains uncompromised. During this step excessive bleeding through the paravaginal varices or branches of the vaginal artery can occur, thus particular caution is needed. When the vaginal dissection has been completed down to the pelvic floor (Level 3 according to De Lancey) (4), the laparoscopy ends with removal of the robot and repositioning of the patient in the lithotomy position. Finally, we perform a circular vaginal incision just above the hymen and the uterus with the vagina are removed through the vaginal canal. The rectovaginal and vesicovaginal fascia are approximated using a continuous suture and the introitus is closed using braided, absorbable suture.

#### Postoperative care

The patient is given the usual pain medication according to the anesthesiological protocols and is mobilized a few hours after surgery. Standard postoperative care is being offered and in most cases the Folley catheter is removed on the next day. The patient can be dismissed on the 2nd or 3rd postoperative day and the follow up control is planned 4 weeks later. Intraoperativ complications are being documented using the Clavien-Dindo classification (5).

## Results

Eighteen individuals underwent robotic assisted laparoscopic hysterectomy (with salpingoophorectomy) and colpectomy in our Instituion between November 2022 and February 2025. The patients had a median age of 28 years and a mean BMI of 23.5 ± 3.8 Kg/m^2^. Mean operating time was 175 ± 25 min (median 180), mean blood loss was 219 ± 142 ml (median 200) and mean hospital stay was 4.6 ± 5.9 days (median 3). Mean robotic operating time was 102 ± 15 and vaginal operating time 73 ± 11 min respectively. Five cases (27%) of urinary retention postoperatively occured (Clavien-Dindo I): three of them resolved within the hospital stay after repeated bladder catheterisation and two required self-intermittent catheterization for two and three weeks respectively. Two (11%) urinary tract infections (Clavien-Dindo II) were diagnosed (at the third and fifth day postoperative day respectively), which required use of antibiotics. One (5%) bladder perforation (Clavien-Dindo I) occurred at the time of surgery, which was resolved intraoperatively with a two layered bladder suture and indwelling catheter for 10 days after surgery. One patient (5%) developed a lower extremity compartment syndrome, which required multiple revisions under general anaesthesia, including fasciotomy and decompression (Clavien-Dindo IIIb). A detailed overview of the complications is being shown in Table 1.

**Table 1:** Complications and their management in our cohort of 18 patients, who underwent robotic assisted laparoscopic colpectomy.

Complication	*N* (%)	Clavien-Dindo Classification	Management
Urinary Retention	5 (27%)	I	- 3 resolved within 4 days with repeated catheterization - 2 required intermittent self catheterization for 2 and 3 weeks respectively
Lower extremity compartment syndrome	1 (5%)	IIIb	Repeated surgical fasciotomy and wound decompression
Bladder injury	1 (5%)	I	2-layer suture of the bladder wall, cystoscopy
UTI	2 (11%)	II	Oral antibiotics within the first week post intervention

UTI, urinary tract infection.

## Discussion

Colpectomy (also called vaginectomy) at the time of hysterectomy in transgender people is regarded as a challenging procedure. Removal of the vagina—as described in studies for colpectomy for pelvic organ prolapse or malignant disease—requires a meticulous dissection near the paravesical and pararectal spaces as well as a broad understanding of the vaginal and paravaginal anatomy and connection to adjacent structures (6). Colpectomy in trans men is even more challenging: many of these patients are already under hormonal treatment, which can decrease the thickness of the vaginal epithelium (7), thus making the vaginal but also the surrounding tissues fragile and difficult to manipulate during dissection. Moreover, many young trans men who seek surgical care are presented as virgins or nulliparous, thus offering less optimal exposure of the pelvic organs than, for example, cis women with pelvic organ prolapse and vaginal laxity due to previous deliveries.

Although colpectomy has been described as a laparoscopical, robotic or vaginal method, it has not been standardized yet. A review from 2022 revealed six studies reporting on transvaginal colpectomy and only a single publication on robotic assisted laparoscopic colpectomy (8). Groenman et al. describe a robotic approach, which first detaches the uterus from the vaginal vault completely and consequently continues with the vaginal dissection, while simultaneously a second surgeon dissects the vagina from the perineum until meeting the dissection point from above (9). During this technique, as shown in their accompanying video, the tension applied on the vaginal wall is minimal and only partial—the robotic surgeon uses one forceps to consecutively pull at the point of dissection. The method that is described in the current paper, uses a uterovaginal manipulator to move the vaginal walls and create tension, not only to the vaginal walls as a whole, but also to the surrounding tissues, like the bladder pillars, paravaginal suspension and ureterovesical junction—this is obvious in the Supplementary Video S1. This facilitates the dissection of the right plane. Another benefit is the lack of a second surgical team, since only one assistant is needed between the patients’ legs to manipulate the device. Another advantage of using the proposed (or similar) uteromanipulator device is the fact that it provides a steady surface upon which the dissection of the vagina from the bladder and rectum is facilitated significantly.

According to the literature, the complication rates of colpectomy are quite high: perineal hamatoma 10.5%, excessive bleeding up to 30% (10), abscess formation 4% (11), urinary retention 16.7% (9). The most recent publication on colpectomy in transgender men, which is also the one with the largest cohort, was published in 2024 by Nikkels et al. Although they compared two non/homogenous groups (140 who underwent robotic assisted hysterectomy and colpectomy vs. 170 individuals with previous hysterectomy who underwent vaginal colpectomy only), the found that the one-stage approach, while more extensive in operating time, showed less blood loss and hospital stay as well as lower risk of intraoperative complications as the vaginal colpectomy alone (12). However both groups presented with high complications rate: 45% vs. 54.7% overall complications and 7.1% vs. 18% ≥ Clavien-Dindo IIIa complications respectively. At the time of writing there are only four publications that assess the laparoscopic colpectomy in transgender people, three robotic assisted and one with conventional laparoscopy, a detailed list can be seen on Table 2.

**Table 2:** Published studies on laparoscopic (conventional or robotic-assisted) colpectomy including complications.

Publication (year)	*N*	Method of colpectomy	OR time (minutes)	Complications
Nikkels et al. (2024) (12)	140^a^	Robotic assisted laparoscopy Removal of the vaginal epithelium using monopolar scissors (similar to Groenman et al)	Median 176 (153–257)	- Urethra injury 1/140 (0.7%) - Urinary retention 24/140 (17.1%) - Urinary tract infection 11/140 (7.9%) - Hemorrhage requiring re-surgery 6/140 (4.3%)
Groenman et al. (2017) (8)	36	Robotic assisted laparoscopy Removal of uterus and adnexa using a uterine mobilizer, followed by Removal of the vaginal epithelium using monopolar scissors	Median 230 (197–278)	- Postoperative bleeding with readmission 1/36 (2.8%) - Urinary tract infection 2/36 (5.6%) - Urinary retention needing catheter 6/36 (16.7%)
Gomes da Costa et al. (2015) (19)	23	Laparoscopic colpectomy ‘In toto’ removal of uterus, adnexa and vagina, using a uterine manipulator Use of bipolar forceps, ligation of vaginal arteries Subsequent phalloplasty	Mean 155 (±42)	- Postoperative hematoma 2/23 (8.7%) - 1 requiring second look laparoscopy - 1 resolved with antibiotics - Urinary retention 1/23 (4.3%)
Ergeneli et al. (1999) (20)	8	Laparoscopic assisted vaginal colpectomy Two surgeons performing simultaneously laparoscopically and vaginally Subsequent phalloplasty	Average 140	- Bladder perforation 1/8 (12.5%) - Deep vein thrombosis 2/8 (25%)

^a^ 140 individuals in the laparoscopic group.

Our small cohort reveals similar numbers regarding the complications. Our first major complication was the development of a compartment syndrome of the lower limbs bilaterally. This was the only one of our eighteen patients on whom a concomitant mastectomy was performed. Additionally, the patient had an hematological medical history with a non-specific plattelet dysfuntion, and showed unusual high blood loss during surgery. The combination of these two factors resulted in an excessively prolonged total operating time which probably triggered the compartment syndrome. The patient had multiple revisions on the lower limbs including fasciotomy and decompression. The second major complication, a bladder injury, occured in a virgin patient with excessively narrow vaginal space and atrophy, something that made dissection of the bladder particularly difficult during the vaginal part of the procedure.

The most common complication in our cohort is urinary retention (27%). This is on par with previous published studies on colpectomy, although a bit higher. Nikkels et al. in 2019 reported urinary retention of 13% of patients who underwent transvaginal colpectomy (13), whereas in their robotic laparoscopic cohort from 2024 the urinary retention rate was 17.1% (12). Groenmann et al. in 2017 report a 16.7% rate of urinary retention (9). The fact that our rates were higher may be related to the fact that we have a strict protocol for residual urine measurement after major pelvic surgery, which comprises of pro-active control of micturition after removal of the indwelling catheter and residual volume measurement, putting a cut-off at 150 ml. Previous authors did not describe a similar action, so it could be possible that covert urinary retention may have been undiagnosed.

**Figure 3:**
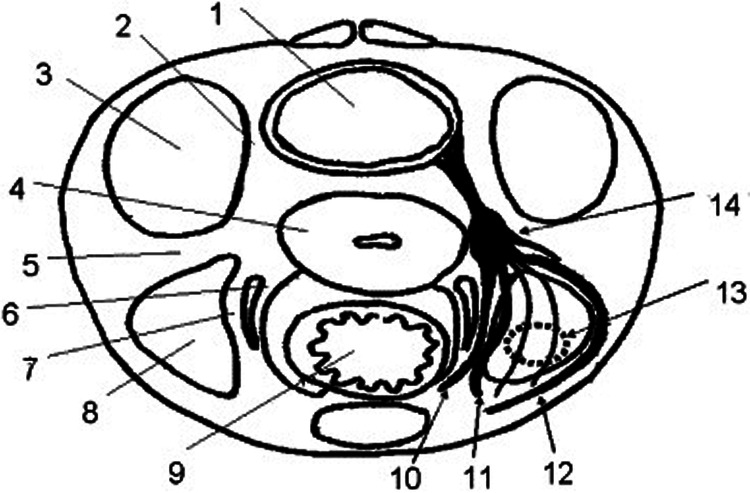
Relationship between parasympathetic and sympathetic fibers in the posterior aspect of the pelvis. (1) Bladder, (2) vesicouterine ligament, (3) paravesical space, (4) uterus, (5) cardinal ligament, (6) medial layer of uterosacral ligament, (7) lateral layer of uterosacral ligament, (8) pararectal space, (9) rectum, (10) hypogastric nerve, (11) first group of parasympathetic nerve, (12) second group of parasympathetic nerve, (13) third group of parasympathetic nerve, and (14) inferior hypogastric plexus [used with permission from Raspagliesi et al. (17)].

Urinary retention and bladder dysfunction in general seems to be a common sequela of pelvic surgery and also corresponds to the extension of the procedure: up to 55% of female patients with cervical cancer present with some degree of urinary retention after radical hysterectomy (14). Urogynecological procedures also show rather high rates of postoperative bladder dysfunction: numbers vary from 4.3% to 26.4% (15, 16). What these procedures have in common, is the proximity of the dissection plane to the cardinal and uterosacral ligaments, structures that are anatomically related to the course of the hypogastric plexus and the parasympathetic nerves of the pelvis (17). Subsequently, techniques that minimize injury to these structures are proven to offer better postoperative bladder function (18). In the case of colpectomy, whether laparoscopic, robotic or transvaginally, the unavoidable dissection of the cardinal and uterosacral ligaments has to be performed with utmost consideration regarding this aspect and as close to the cervix and vaginal dome as possible (Figure 3). According to our experience, every patient after colpectomy should be closely monitored for signs or symptoms of bladder dysfunction, since this could be a reason for prolonged hospital stay or patient discomfort.

This paper comes not without limitations. Firstly, we present a small cohort of patients, which makes generalizabilty of the results rather difficult. Secondly, there is no control group to compare the method that we present. However, this is not the scope of this paper. Since most of the published reports present rather small case series and as shown from previous authors, the learning curve for performing a colpectomy seems to be rather steep (9), there is an urgent need for proper education of surgeons on how to perform this procedure. Accordingly, the present paper offers a precise description of the technique as well as an instructional video as Supplementary Material, in order to provide informative insight into the procedure's critical points. Larger studies are needed to help assess and standardize the colpectomy method in trans men seeking gender affirming surgery.

## Conclusion

As shown both from the literature and from the results of this paper, colpectomy as part of gender affirming surgery in trans men is a technically demanding surgical procedure with a high complication rate. Most complications are minor and self-limiting but major complications can occur, which may require surgical revision or other treatment. Patients should be accordingly counseled and prepared, while surgeons should obtain tenuous educational support for this procedure.

## Data Availability

The raw data supporting the conclusions of this article will be made available by the authors, without undue reservation.
